# *ZAT10* plays dual roles in cadmium uptake and detoxification in *Arabidopsis*

**DOI:** 10.3389/fpls.2022.994100

**Published:** 2022-08-30

**Authors:** Fengfeng Dang, Yajing Li, Yanfeng Wang, Jinhui Lin, Shenxiu Du, Xinyang Liao

**Affiliations:** ^1^State Key Laboratory for Conservation and Utilization of Subtropical Agro-Bioresources, College of Life Sciences, South China Agricultural University, Guangzhou, China; ^2^Shaanxi Key Laboratory of Chinese Jujube, Yan’an University, Yan’an, China; ^3^College of Agriculture, Fujian Agriculture and Forestry University, Fuzhou, China

**Keywords:** *ZAT10*, Cd stress, uptake, detoxification, FIT, *Arabidopsis*

## Abstract

Cadmium (Cd) is a harmful heavy metal that is risky for plant growth and human health. The zinc-finger transcription factor ZAT10 is highly conserved with ZAT6 and ZAT12, which are involved in Cd tolerance in plants. However, the definite function of ZAT10 in Cd tolerance remains uncertain. Here, we demonstrated that ZAT10 negatively regulated Cd uptake and enhanced Cd detoxification in *Arabidopsis.* The expression of *ZAT10* in plants is induced by Cd treatment. The *zat10* mutant plants exhibited a greater sensitivity to Cd stress and accumulated more Cd in both shoot and root. Further investigations revealed that ZAT10 repressed the transcriptional activity of *IRT1*, which encodes a key metal transporter involved in Cd uptake. Meanwhile, ZAT10 positively regulated four heavy metal detoxification-related genes: *NAS1*, *NAS2*, *IRT2*, and *MTP3*. We further found that ZAT10 interacts with FIT, but their regulatory relationship is still unclear. In addition, ZAT10 directly bound to its own promoter and repressed its transcription as a negative feedback regulation. Collectively, our findings provided new insights into the dual functions of ZAT10 on Cd uptake and detoxification in plants and pointed to *ZAT10* as a potential gene resource for Cd tolerance improvement in plants.

## Introduction

Cadmium (Cd) is toxic to plants and poses a significant risk to food production and food safety. The accumulation of cadmium in agricultural soils has increased as a result of human activities and modern industrial practices (Antoniadis et al., 2017; Geng et al., 2019; Wang et al., 2019; Palansooriya et al., 2020). Many studies have shown that Cd inhibits chlorophyll synthesis and root development, leading to new leaf chlorosis, root elongation inhibition, and even plant death (Verbruggen et al., 2009; Bae et al., 2016; Dang et al., 2019). Plants have evolved several potential mechanisms to cope with Cd stress, such as avoiding cadmium toxicity by limiting Cd uptake and enhancing Cd tolerance through accumulation, storage, and immobilization of Cd elements in certain plant tissues (El Rasafi et al., 2020).

However, no specific transporter or channel for the uptake of Cd into plant cells has been reported. The uptake of Cd is mostly attributed to cationic transporters or channels, such as the Iron Regulated Transporter 1 (IRT1), Zinc-regulated/Iron-regulated transporter-like family Proteins (ZIPs), Natural Resistance-Associated Macrophage Protein family (NRAMP), and metal pump ATPase (Gallego et al., 2012; Antoniadis et al., 2017; Zheng et al., 2018; Spielmann et al., 2020). In our previous study, we pointed out that Cd exposure caused Fe deficiency-induced chlorosis in emerging leaves of pepper plants (Dang et al., 2019). Several studies have shown that IRT1 is involved in the regulation of Cd uptake from soil (Lombi et al., 2002; Vert et al., 2002; Fan et al., 2014). These studies suggested that Cd competed with Fe in the process of root uptake of metal elements. This competition, in turn, exacerbates the Fe deficiency response in plants and results in the up-regulation of *IRT1* transcript level, further leading to more Cd uptake. In addition, the FER-like iron deficiency-induced transcription factor (FIT), a basic helix loop helix (bHLH) transcription factor, is the pivotal regulator of Fe-deficiency responses and Fe homeostasis in *Arabidopsis*. It has been reported that FIT interacts with various proteins, such as bHLH038, bHLH039, bHLH100, bHLH101, bHLH18, bHLH19, bHLH20, bHLH25, and EIN3/EIL1, to regulate the expression of *IRT1* and thus the uptake of Cd (Yuan et al., 2008; Lingam et al., 2011; Zhu et al., 2013; Wu and Ling, 2019).

On the other hand, plants up-regulate the expression of genes relevant to heavy metal sequestration and chelation to prevent heavy metal toxicity, such as the vacuolar membrane-localized metal transporters *Heavy Metal Associated 3* (*HMA3*), *Heavy Metal Associated 4* (*HMA4*), *Metal Tolerance Protein 1* (*MTP1*), *Metal Tolerance Protein 3* (*MTP3*), *Iron Regulated Gene 2* (*IREG2*), and the intracellular vesicle membrane protein *IRT2* (Arrivault et al., 2006; Morel et al., 2009; Vert et al., 2009; Wu et al., 2012; Yao et al., 2018). Previous studies have shown that these genes participate in cytoplasmic detoxification through transporting heavy metals into vacuoles and vesicles of cells. It was also found that *Nicotianamine Synthase* (NAS) confers Cd tolerance by increasing NA content (Kim et al., 2005; Klatte et al., 2009). The *bHLH104* has been shown to participate in Cd tolerance through regulating heavy metal sequestration and detoxification-associated genes, such as *MTP3*, *HMA3*, *IREG2*, and *NAS4* (Yao et al., 2018). Likewise, the zinc-finger transcription factor *ZAT6* is induced by Cd stress and positively regulates the expression of *GSH1* and PC biosynthesis-related genes, which consequently enhances Cd tolerance (Chen et al., 2016). In addition, besides affecting Cd uptake, FIT also regulates Cd tolerance by interacting with bHLH38 or bHLH39 to form FIT/bHLH38 and FIT/bHLH39 heterodimers. These modules increase the expression of *MTP3*, *HMA3*, *IREG2*, *IRT2*, *NAS1*, and *NAS2*, to enhance Cd sequestration in plant cells (Wu et al., 2012). However, while these studies have identified some of the major genes involved in Cd detoxification and tolerance in plants, a more comprehensive understanding of the transcriptional networks that regulate Cd tolerance is still required.

The zinc-finger transcription factor ZAT10 is highly conserved with ZAT6 and ZAT12, which are involved in the responses to Cd stress and abiotic stress in plants (Mittler et al., 2006; Opdenakker et al., 2012; Shi et al., 2014; Chen et al., 2016). However, the definite function of ZAT10 in Cd tolerance is still unknown. In the present study, we revealed the novel function of ZAT10 in response to Cd stress in plants. ZAT10 negatively regulated Cd uptake by repressing the expression of *IRT1* and enhanced Cd detoxification by promoting the expression of Cd sequestration genes.

## Materials and methods

### Sequences alignment and phylogenetic analysis

The amino acid sequences used for sequence alignment and phylogenetic analysis were obtained from NCBI protein database. The amino acid sequence alignment was conducted using the DNAMAN software. The phylogenetic tree was performed by MEGA 10 software using the maximum likelihood method with default settings.

### Plant material, growth conditions

The *Arabidopsis thaliana* ecotype Col-0 was used as the wild type in this study. The *ZAT10* T-DNA insertion line (*zat10*, SALK_054092C) obtained from the *Arabidopsis* Biological Resource Center (ABRC^1^) was used (Alonso et al., 2003). *ZAT10* primers and the T-DNA left-border primer (LBb1.3) were used for the identification of T-DNA lines (Supplementary Table 1). Surface-sterilized seeds were exposed to 4°C for 2 days before germinated on culture. For phenotype assays, WT and *zat10* mutant seeds were sown on 1/2 Murashige and Skoog (MS) medium containing 0.8% agar (Sigma, no. A1296) with 0, 30, 60, or 90 μM CdSO_4_ for 9 days. For gene expression analysis under Cd stress, about 12 seedlings were cultured in 1 ml of the 1/2 MS liquid medium in the six-well tissue culture plate (Sangon Biotech, Shanghai, China) for 5 days. Then, seedlings were washed three times with 1 mL 1/2 MS medium and were transferred to 1/2 MS liquid medium containing the indicated concentration of CdSO_4_ for a specific period of time. Seedlings were harvested for RNA extraction using RNAprep Pure Plant Kit (Tiangen Biotech, Beijing, China). *Arabidopsis* seedlings were grown at 22 ± 2°C in the growth chamber under a 16 h light/8 h dark cycle with a light intensity of about 100 μmol photons m^–2^s^–1^. *Nicotiana benthamiana* was used for transient expression experiments in this study, and plants were grown in growth room at 28 ± 2°C under a 16 h light/8 h dark cycle.

### Molecular cloning and plasmid construction

To generate complementary lines of *zat10* mutant, a genomic DNA fragment of *ZAT10* with its own promoter (∼2,000 bp upstream of the ATG) was amplified using primers listed in Supplementary Table 1 and cloned into *Sac*I and *Sal*I sites of pCAMBIA1300 vector without *35S* promoter. *Agrobacterium*-mediated *Arabidopsis* transformation was performed by the floral dip method.

For molecular analysis, the full-length coding fragments of *ZAT10*, *ZAT12*, *FIT*, and the promoters of *ZAT6*, *ZAT10*, *FIT*, *IRT1*, *ZIP1*, *ZIP3*, *ZIP4*, *ZIP5*, *ZIP9*, *IREG1*, *IRGE2*, *NAS1*, *NAS2*, *MTP3*, and *HMA3* were amplified by PCR using the specific primers listed in Supplementary Table 1. And then these fragments were cloned into the *pCE-Zero* vector using the ClonExpress Ultra One Step Cloning Kit (Vazyme, Nanjing, China) and sequenced by Sangon Biotech (Shanghai, China). Then the *ZAT10* coding region was inserted into the plant expression vector (*35S-GFP-NOS* plasmid) to generate the *35S:ZAT10-GFP* construct. Promoters of target genes were fused into the plant reporter vector (*35S:REN-NOS-promoter:LUC-NOS* plasmid) to generate the *35S:REN-promoter:LUC* construct.

### Histochemical staining

Hydrogen peroxide (H_2_O_2_) and superoxide radicals (O_2_^–^) were analyzed using 3, 3′-diaminobenzidine (DAB) and NitroBlue Tetrazolium (NBT), respectively, as described previously (Dang et al., 2013, 2019; Shi et al., 2013). In brief, the seedlings were immersed in DAB solution (pH 3.8, 1 mg/ml) or NBT solution (1 mg/ml) at 25°C for 12 h. The seedlings were then completely bleached by 70% ethanol (v/v) and photographed by a Stereo Microscope (Leica, Wetzlar, Germany).

### Measurements of cd and hydrogen peroxide content

Measurements of Cd and H_2_O_2_ content were performed as described previously (Dang et al., 2019; Ding et al., 2021, 2022). In brief, 7-day-old *Arabidopsis* seedlings were transferred to 1/2 MS medium containing 30 μM CdSO_4_ for 3 and 5 days, then samples were harvested and used for Cd and H_2_O_2_ measurements. For Cd measurement, samples were analyzed *via* the inductively coupled plasma-atomic emission spectrometer (IRIS/AP Optical Emission Spectrometer, Thermo Scientific Pierce, Waltham, MA, United States). For H_2_O_2_ measurement, approximately 80 mg of samples were analyzed by an Amplex Red H_2_O_2_ assay Kit (Invitrogen, Thermo Scientific Pierce, Waltham, MA, United States).

### Yeast one-hybrid and yeast two-hybrid assays

For the Y1H assay, the AD fusion construct (*pB42AD*) was co-transformed into yeast (EGY48) with the *pLacZ2*μ construct containing a specific promoter, and then were selected on SD/-Trp-Ura agar plates for 72–96 h at 30°C. Subsequently, transformants of yeast were carried out using the selective medium containing raffinose, galactose, and X-gal (Amresco, Solon, OH, United States). For the Y2H assay, the AD-fusion (*pGADT7*) was co-transformed into yeast (AH109) with the BD-fusion (*pGBKT7*). Yeast (Y2H) transformants were then selected on selective medium (SD-LWHA) with or without 10 mM 3-amino-1,2,4-triazole (3-AT).

### Electrophoretic mobility shift assay

For the EMSA assay, the CDS fragment of *ZAT10* was cloned into the *pMAL*-*c4X* vector. The recombinant plasmid or empty vectors were then transformed into *Escherichia coli* BL21. 0.4 mM IPTG was added to induce the fusion proteins of MBP-ZAT10 for 16–20 h at 16°C with 200 rpm shaking in liquid LB medium. The MBP-ZAT10 protein was purified using Amylose Resin (NEB, Beijing, China). Briefly, the protein-bound beads were washed three times with CB buffer (20 mM Tris–HCl, 100 mM NaCl, 10 mM EDTA, pH 7.4). The reaction mixture was then incubated for 3 h at 4°C. Then, the proteins were eluted with elution buffer (20 mM Tris–HCl, 100 mM NaCl, 10 mM EDTA, 0.5 mM maltose, pH 7.4). Subsequently, the MBP-ZAT10 protein (2 μg) was incubated with probes using EMSA/gel-shift binding 5 × Buffer (Beyotime Biotechnology, Shanghai, China) in 20 μL reaction mixtures at 25°C for 30 min. To generate the Cy5-labeled probes, the promoter fragment of *ZAT10* was synthesized (Supplementary Table 1). After that, samples were separated by 12% native polyacrylamide gels. Then the gel was visualized using a LI-COR Odyssey Infrared Imaging System to detect the fluorescent label (LI-COR, Lincoln, NE, United States).

### Transient expression assays

Transient expression assays in *Arabidopsis* mesophyll protoplasts were performed as described previously (Yoo et al., 2007). In brief, the plasmids were isolated and purified using the EndoFree Maxi Plasmid Kit (Tiangen Biotech, Beijing, China) according to the manufacturer instructions. And then analysis of relative luciferase activity driven by indicated promoter was carried out in mesophyll protoplasts. The protoplasts were transfected with 20 μg *35S:REN*-*promoters:LUC* and 20 μg *p19-ZAT10-GFP or p19-GFP* plasmids and then incubated in WI buffer for 10 h. To investigate the functional relationship between ZAT10 and FIT protein, the protoplasts were transfected with 20 μg *35S:REN*-*promoters:LUC* plasmids, and 10 μg of *p19-ZAT10-GFP, p19-FIT-GFP*, or *p19-GFP* plasmids in different combinations to reach a total of 20 μg per transfection reaction, and then incubated in WI buffer for 10 h. The activity of firefly and control Renilla luciferase (REN) was analyzed by the Dual-Glo Luciferase Assay System (Promega, Beijing, China) after evaluating the GFP signal using fluorescence microscopy. The firefly luciferase activity was normalized to the REN luciferase activity (LUC/REN).

Transient transcription assays of *N. benthamiana* plants were performed as described (Liu et al., 2019a,b). In brief, the promoter fragments of *ZAT6*, *ZAT10*, *FIT*, *IRT1*, *ZIP1*, *ZIP3*, *ZIP4*, *ZIP5*, *ZIP9*, *IREG1*, *IRGE2*, *NAS1*, *NAS2*, *MTP3, or HMA3* were cloned into the *HindIII* and *BamHI* digested *pGreen*-*0800*-*LUC* vector, and the CDS fragment of *ZAT10* was inserted into *BamHI* and *HindIII* digested *pGreenII 62*-*SK* vector. These constructs were transformed into *Agrobacterium* EHA105 (pSoup), and leaves of 6 to 8-week-old *N. benthamiana* plants were infiltrated with suspension of *Agrobacterium* containing the indicated constructs (*OD*_600_ = 0.5), and then plants were transferred to a growth room for 48 h at 28 ± 2°C. The firefly luciferase luminescence was photographed after infiltrating with 1 mM luciferin (88294, Thermo Scientific Pierce, Waltham, MA, United States), and quantified using the Night SHADE LB985 system (Berthold, Stuttgart, Germany).

### Bimolecular fluorescence complementation assay

Bimolecular fluorescence complementation assays were performed on *Arabidopsis* protoplasts. In brief, the coding fragment of *FIT* was inserted into the *p19-YFPN* vector, and the coding fragments of *ZAT10* and *ZAT12* were inserted into the *p19-YFPC* vector, respectively. Subsequently, constructs were co-transformed into *Arabidopsis* protoplasts and incubated for 10 h. YFP fluorescence signals were captured using a fluorescence microscope (Nikon, Tokyo, Japan).

### Split luciferase complementation imaging assay

As described previously, the LCI assay was performed on *N. benthamiana.* In brief, the coding fragments of *ZAT10* and *ZAT12* were inserted into the *KpnI* and *SalI* digested *pCAMBIA-nLUC* vector, respectively. The coding sequence of FIT was inserted into the *KpnI* and *SalI* digested pCAMBIA-cLUC vector. Then the *ZAT10-nLUC*, *ZAT12-nLUC*, *cLUC-FIT*, *nLUC*, and *cLUC* constructs were transformed into *Agrobacterium* EHA105 as indicated combinations. The suspension (*OD*_600_ = 0.5) of *Agrobacterium* containing the indicated constructs was infiltrated into the leaves of 6 to 8-week-old *N. benthamiana* plants using a needleless syringe. After infiltration, plants were grown under 16 h light/8 h dark for 36–48 h. Leaves were then infiltrated with 1 mM luciferin (88294, Thermo Scientific Pierce, Waltham, MA, United States), and the LUC signal was captured using the LB985 Night SHADE system (Berthold, Stuttgart, Germany) with a 30 s exposure.

### Gene expression analysis

For gene expression assays, RNA was extracted using the RNAprep Pure Plant Kit (Tiangen Biotech, Beijing, China). The TaKaRa PrimeScript RT-PCR Kit (TaKaRa Bio, Beijing, China) was used to synthesize cDNA following the reverse transcription protocol. Expression levels of genes were analyzed by the CFX96 Real-Time PCR System (Bio-Rad, Hercules, CA, United States). *Arabidopsis UBIQUITIN 10* (*UBQ10*) was used as an internal control. Primers for qRT-PCR assay were listed in Supplementary Table 1.

### Statistical analysis

All experiments were performed using three biological replicates. All the data were analyzed by Student’s *t*-test or by Tukey’s multiple comparison test. Statistically significant differences were indicated by asterisks or different letters.

### Accession numbers

Sequence data from this article can be found in the *Arabidopsis* Information Resource (TAIR^2^) or GenBank/EMBL data libraries under the following accession numbers: *ZAT10* (AT1G27730), *ZAT6* (AT5G04340), *ZAT12* (AT5G59820), *ZF1* (AT5G67450), *ZF2* (AT3G19580), *ZF3* (AT5G43170), *FIT* (AT5G59820), *IRT1* (AT4G19690), *ZIP1* (AT3G12750), *ZIP2* (AT5G59520), *ZIP3* (AT2G17790), *ZIP4* (AT1G10970), *ZIP5* (AT1G05300), *ZIP9* (AT4G33020), *IRT2* (AT4G19680), *NAS1* (AT5G04950), *NAS2* (AT5G56080), *IREG1* (AT2G38460), *IREG2*(AT5G03570), *MTP3* (AT3G58810), *HMA3* (AT4G30120), *UBQ10* (AT4G05320), *GmZAT10* (Glyma.04G044900), *SlZAT10* (Solyc04g077980), *CaZAT10* (CA00g39910), *OsZAT10* (LOC_Os12g39400), and *ZmZAT10* (GRMZM2G069176).

## Results

### The expression of *ZAT10* is induced by cadmium treatment

**FIGURE 1:**
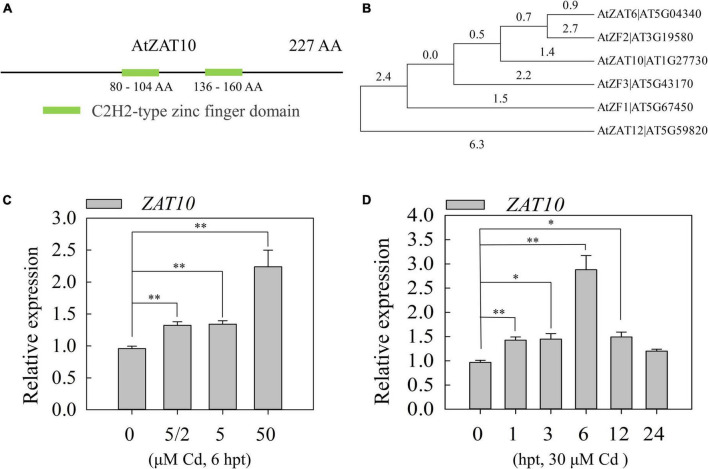
Molecular characterization of ZAT10 and the expression patterns of *ZAT10* under cadmium stress. **(A)** The sites of the predicted two C2H2-type zinc-finger domains in the ZAT10 protein. **(B)** Phylogenetic tree of ZAT10 and its homologous members in *Arabidopsis*. **(C,D)** Relative expression levels of *ZAT10* in 5-day-old wild type seedlings **(C)** under CdSO_4_ (0, 2.5, 5, and 50 μM) treatment for 6 h and **(D)** at 0, 1, 3, 6, 12, 24 h post treatment (hpt) with 30 μM CdSO_4_. Means ± SD, *n* = 3; *P < 0.05 or ^**^P < 0.01, Student’s *t*-test.

ZAT10, a zinc-finger transcription factor, contains two C2H2-type zinc-finger domains, which are highly conserved in ZAT10 sequences from diverse plant species (Figure 1A and Supplementary Figure 1). The phylogenetic analysis of ZAT10 and its homologs in *Arabidopsis* showed that ZAT10 has a high degree of similarity and conservation with ZAT6 and ZAT12, which are involved in the responses to Cd stress in plants (Figure 1B; Opdenakker et al., 2012; Chen et al., 2016). Therefore, we hypothesized that *ZAT10* has a potential function in the regulation of Cd tolerance in plants. To verify this hypothesis, we first investigated whether *ZAT10* responds to Cd stress in plants. The qRT-PCR analysis revealed that the transcript level of *ZAT10* was induced in *Arabidopsis* seedlings under different concentrations of Cd treatment (Figure 1C). We also examined the temporal expression pattern of *ZAT10* in response to Cd exposure. As shown in Figure 1D, *ZAT10* was a fast-responding gene that was induced by 1 h of Cd exposure and peaked at 6 h, followed by a significant decrease after 12 h of treatment. These results indicate that *ZAT10* is involved in regulating the response to Cd stress in plants.

### Loss-of-function of *ZAT10* lead to increased cadmium sensitivity

**FIGURE 2:**
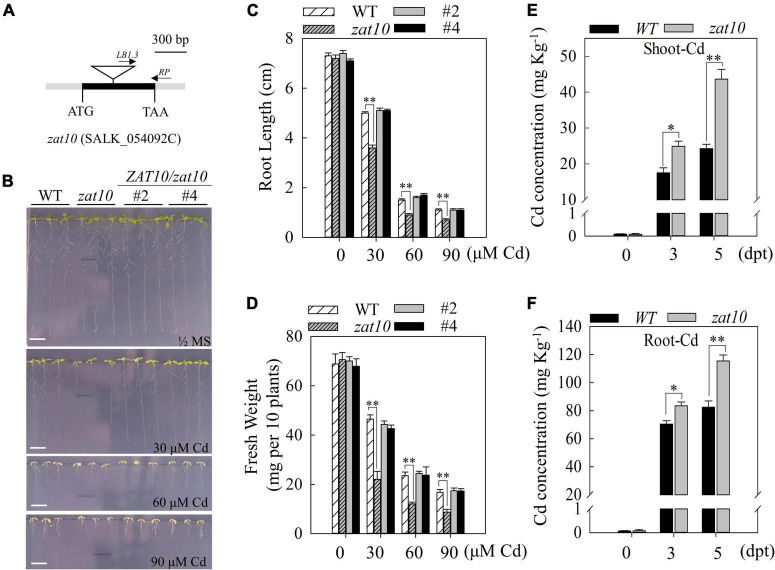
The *zat10* loss-of-function mutant is sensitive to Cadmium stress. **(A)** Schematic diagram of *T-DNA* insertion site on the locus of *ZAT10* in the *zat10* (SALK_054092C) mutant. **(B)** Phenotypic analysis of the wild type, *zat10* mutant and *ZAT10pro:ZAT10/zat10* complementary lines (*ZAT10/zat10 #2*, *#4*) under Cd stress. Nine-day-old seedlings grown on 1/2 MS medium without or with CdSO_4_ (30, 60, and 90 μM). Bar = 1 cm. Three independent experiments were done with similar results. **(C,D)** Root length **(C)** and fresh weight **(D)** of the wild type, *zat10* mutant, and *ZAT10/zat10 #2, #4* lines under the condition described in B. Means ± SD, *n* = 3; ^**^P < 0.01, Student’s *t*-test. **(E,F)** Cd concentration in shoot **(E)** and root **(F)** of the wild type and *zat10* mutant under Cd stress. Seven-day-old seedlings grown on 1/2 MS medium were transferred to 1/2 MS medium without or with 30 μM CdSO_4_ for 0, 3, and 5 days. Means ± SD, *n* = 3; *P < 0.05 or **P < 0.01, Student’s *t*-test.

To characterize the function of *ZAT10* in plant Cd-tolerance, we obtained the *Arabidopsis* T-DNA insertional mutant *zat10* (SALK_054092C) from the SALK T-DNA collection (Figure 2A; Mittler et al., 2006) and generated complementary lines expressing *ZAT10pro:ZAT10* in the *zat10* background. Seeds of the wild type, *zat10* mutant and two independent complementary lines were germinated on 1/2 MS agar plates with or without supplementary 30, 60, or 90 μM Cd for 9 days. Plants grown on 1/2 MS without Cd displayed similar plant size and root length, whereas those with Cd treatment showed suppressed plant growth and root elongation (Figures 2B–D). Compared to the wild type, the *zat10* mutants were more sensitive to Cd stress (Figures 2B–D). Meanwhile, both complementary lines showed comparable root length and biomass as the wild type, demonstrating that *ZAT10* is functional in Cd tolerance and can rescue the *zat10* phenotype under Cd exposure (Figures 2B–D). We further tested the Cd content in the shoot and root of plants that were exposed to Cd. The wild-type and *zat10* mutant plants grown on 1/2 MS agar plates without Cd for 7 days were transferred to 1/2 MS agar plates with 30 μM Cd. At indicated days post treatment (DPT), Cd concentration in the shoot and root was measured, respectively. The results showed that more Cd was accumulated in the shoot and root of *zat10* seedlings compared with that in the wild type (Figures 2E,F). These results suggest that *ZAT10* plays an important role in regulating Cd accumulation and tolerance in *Arabidopsis*.

### Cadmium induces more reactive oxygen species in *zat10* plants

**FIGURE 3:**
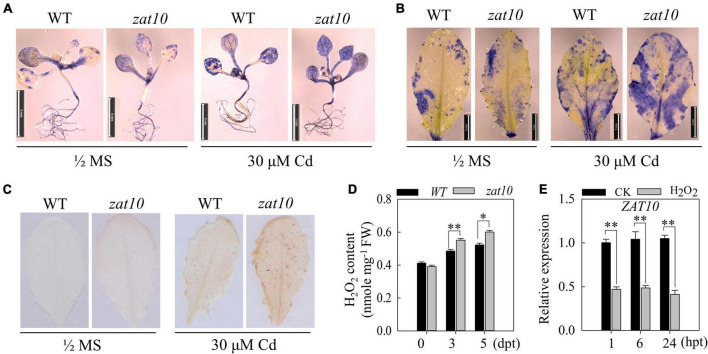
*zat10* plants accumulate more reactive oxygen species under cadmium stress. **(A,B)** The 2-week-old **(A)** and 3-week-old **(B)** wild-type and *zat10* plants were treated with 30 μM CdSO_4_ for 24 h, and then the accumulation of ROS in plants **(A)** and leaves **(B)** was detected by NBT staining. Bar = 5 mm. **(C)** Accumulation of H_2_O_2_ was detected in the wild type and *zat10* leaves under 30 μM CdSO_4_ treatment for 24 h by diaminobenzidine staining. **(D)** H_2_O_2_ concentration in seedlings of the wild type and *zat10* mutant under Cd stress. Seven-day-old seedlings grown on 1/2 MS medium were transferred to 1/2 MS medium without or with 30 μM CdSO_4_ for 0, 3, and 5 days, and then were harvested for H_2_O_2_ concentration measurements. Means ± SD, *n* = 3; *P < 0.05 or ^**^P < 0.01, Student’s *t*-test. **(E)** Expression of *ZAT10* was analyzed by qRT-PCR in the wild-type plants at 0, 6, and 24 h post treatment (hpt) with 10 μM H_2_O_2_. Means ± SD, *n* = 3; ^**^P < 0.01, Student’s *t*-test.

In general, Cd stress induces the accumulation of reactive oxygen species (ROS), such as superoxide radicals (O_2_⋅^–^), hydrogen peroxide (H_2_O_2_), and hydroxyl radicals (OH⋅) (Tran and Popova, 2013; Perez-Chaca et al., 2014). Thus, we applied NBT staining and DAB staining to detect the accumulation of O_2_⋅^–^ and H_2_O_2_ in seedlings or leaves under Cd stress. The results showed that *zat10* mutant seedlings accumulated much higher levels of O_2_⋅^–^ and H_2_O_2_ than the wild type under Cd stress, while the accumulation levels of O_2_⋅^–^ and H_2_O_2_ in *zat10* mutant and wild type seedlings in the absence of Cd were similar (Figures 3A–C). Quantification of H_2_O_2_ content in the seedlings of wild type and *zat10* plants also confirmed these results (Figure 3D). These results demonstrate that Cd treatment induces a higher level of ROS burst in *zat10* seedlings, suggesting that *ZAT10* is involved in the mitigation of Cd-induced ROS stress in plants. To further investigate whether Cd stress or Cd-induced ROS stress induces the expression of *ZAT10*, we examined the transcript level of *ZAT10* in plants treated with H_2_O_2_. Interestingly, the expression level of *ZAT10* in plants sharply decreased after 1 h of H_2_O_2_ treatment, implying that Cd-induced H_2_O_2_ may rapidly and significantly inhibit the expression of *ZAT10* as a feedback regulation (Figure 3E).

### ZAT10 regulates the expression of heavy metal uptake genes

**FIGURE 4:**
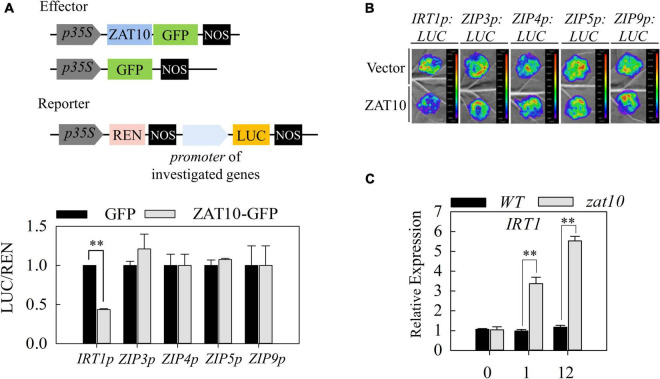
ZAT10 represses the expression of *IRT1*. **(A)** The schematic drawing of vectors used in dual-luciferase assays. Dual-luciferase assays in *Arabidopsis* protoplasts show that additional ZAT10 represses the activity of *IRT1p:LUC*. The REN was used as an internal control. LUC/REN ratio represents the relative activity of the *IRT1*, *ZIP3*, *ZIP4*, *ZIP5*, and *ZIP9* promoters. Means ± SD, n = 3; ^**^P < 0.01, Student’s *t*-test. **(B)** *ZAT10* represses the transcriptional activity of *IRT1* promoter in *N. benthamiana* leaves. Vectors containing *IRT1p:LUC*, *ZIP3p:LUC*, *ZIP4p:LUC*, *ZIP5p:LUC*, or *ZIP9p:LUC* were co-infiltrated with the empty vector or *ZAT10* overexpression vector in *N. benthamiana* leaves as indicated. Images were taken at 48 h after infiltration. At least three replicates were measured with similar results. **(C)** Relative expression of *IRT1* in the wild-type and *zat10* mutant plants at 0, 1, and 12 h post treatment (hpt) with 30 μM CdSO_4_. Means ± SD, *n* = 3; ^**^P < 0.01, Student’s *t*-test.

To reveal how ZAT10 affects plant tolerance to Cd stress, we investigated whether ZAT10, as a transcription factor, regulates some downstream genes that may be involved in the response to Cd stress. It was previously reported that one of the main strategies for plants to defend against heavy metal stress is to reduce the uptake of heavy metals from the soil by the root system (El Rasafi et al., 2020). Therefore, we first investigated the action of ZAT10 on genes related to metal uptake in plants. Considering that some ZIP family members are involved in the uptake of metals ion, both essential metal nutrients and toxic heavy metals (Grotz et al., 1998; Pence et al., 2000; Milner et al., 2013; Zheng et al., 2018; Spielmann et al., 2020), we tested the well-studied metal transporter IRT1, which plays a critical role in plant Cd uptake (Rogers et al., 2000; Vert et al., 2002), and some ZIP genes mainly expressed in roots, such as *ZIP3*, *ZIP4*, *ZIP5*, and *ZIP9* (Zheng et al., 2018). We applied the dual-luciferase assay in *Arabidopsis* protoplasts to examine the transcriptional regulation of ZAT10 on the promoters of these genes. Out of the 5 genes tested, the *IRT1p-LUC* reporter showed a dramatic decrease in luciferase activity when ZAT10 was co-expressed (Figure 4A). Next, a transient expression assay in *N. benthamiana* leaves was carried out to verify this result. Similarly, ZAT10 suppressed the transcriptional activity of the *IRT1* promoter, but had no effect on the other four promoters (Figure 4B). Furthermore, the transcript levels of these genes in the wild type or *zat10* mutant plants under Cd stress were tested by qRT-RCR. Consistently, it was shown that *IRT1*, but not other tested *ZIP* genes, was rapidly induced by Cd treatment in *zat10* mutant plants compared with those in the wild type (Figure 4C and Supplementary Figure 2A). Together, these results suggest that ZAT10 down regulates the expression level of *IRT1* in plants under Cd stress.

### ZAT10 regulates the expression of heavy metal detoxification genes

**FIGURE 5:**
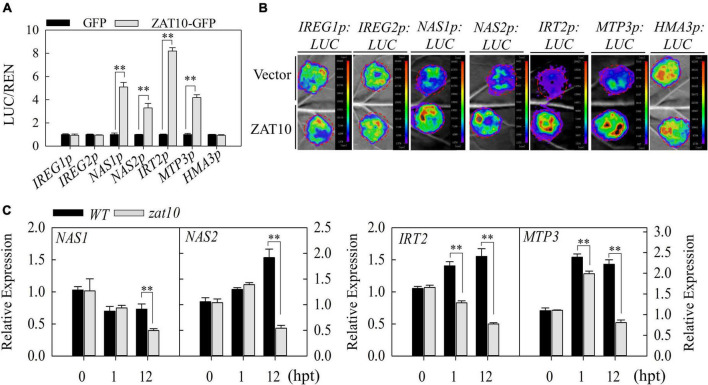
ZAT10 activates the expression of *NAS1*, *NAS2*, *IRT2*, *and MTP3*. **(A)** Dual-luciferase assays in *Arabidopsis* protoplasts show that additional ZAT10 enhances the activity of *NAS1p:LUC*, *NAS2p:LUC*, *IRT2p:LUC*, and *MTP3p:LUC* in protoplast transient expression assays. The REN was used as an internal control. LUC/REN ratio represents the relative activity of the *IREG1*, *IREG2*, *NAS1*, *NAS2*, *IRT2*, *MTP3*, and *HMA3* promoters. Means ± SD, *n* = 3; ^**^P < 0.01, Student’s *t*-test. **(B)** ZAT10 enhances the transcriptional activity of *NAS1*, *NAS2*, *IRT2*, and *MTP3* promoters in *N. benthamiana* leaves. The *IREG1p:LUC*, *IREG2p:LUC*, *NAS1p:LUC*, *NAS2p:LUC*, *MTP3p:LUC*, and *HMA3p:LUC* were co-infiltrated with empty vector or *ZAT10* overexpression vector in *N. benthamiana* leaves as indicated. Images were taken at 48 h after infiltration. At least three replicates were measured with similar results. **(C)** Relative expression levels of *NAS1*, *NAS2*, *IRT2*, and *MTP3* in the wild-type and *zat10* mutant plants at 0, 1, and 12 h post treatment (hpt) with 30 μM CdSO_4_. Means ± SD, *n* = 3; ^**^P < 0.01, Student’s *t*-test.

In addition to reducing the uptake of Cd by roots, plants alleviate the toxicity of heavy metals to cells by chelating or sequestering them in vacuoles and vesicles (El Rasafi et al., 2020). A number of heavy metal detoxification genes have been reported previously. For example, *NAS1* and *NAS2* play a key role in the synthesis of NA, an important chelator in response to heavy metals (Kim et al., 2005; Klatte et al., 2009); *IREG1*, *IREG2*, *IRT2*, *MTP3*, and *HMA3* are all involved in the transport of heavy metals into vesicles and vesicles (Arrivault et al., 2006; Schaaf et al., 2006; Morel et al., 2009; Vert et al., 2009). Thus, we evaluated whether these genes are regulated by ZAT10 through the dual-luciferase assays in *Arabidopsis* protoplasts. The results showed that ZAT10 up-regulated the transcriptional activity of *NAS1p*, *NAS2p*, *IRT2p*, and *MTP3p*, but had no effect on *IREG1p*, *IREG2p*, and *HMA3p* (Figure 5A). Consistent with these results, transient expression assays in *N. benthamiana* leaves showed that co-expression of ZAT10 protein significantly increased the transcriptional activation of the LUC reporter gene driven by *NAS1p*, *NAS2p*, *IRT2p*, and *MTP3p* (Figure 5B), indicating that ZAT10 positively regulates the expression of these genes.

To verify the above results, we further tested the expression levels of these seven genes in wild type and *zat10* plants under Cd stress. qRT-PCR analysis revealed a similar degree of decrease in *NAS1* expression in both wild-type and *zat10* mutant plants after 1 h of Cd treatment, but the expression was further suppressed in *zat10* plants after 12 h of treatment (Figure 5C). Moreover, the expression levels of *NAS2*, *IRT2*, and *MTP3* in the wild type were significantly induced by 1 h of Cd treatment, and the expression levels continued to increase or remained high after 12 h of Cd treatment (Figure 5C). By contrast, the expression of *NAS2* and *MTP3* in *zat10* mutant plants were also up-regulated after 1 h of Cd treatment, but they were considerably decreased after 12 h of treatment (Figure 5C). Meanwhile, *IRT2* in *zat10* mutant plants was inhibited after 1 h of Cd treatment, and this inhibition was enhanced with the duration of treatment (Figure 5C). In addition, in accordance with the transcriptional activation assays, there was no significant difference in the expression levels of *IREG1*, *IREG2*, and *HMA3* between the wild-type and *zat10* mutant plants, either under Cd-free or Cd-exposed conditions (Supplementary Figure 2B). Taken together, these results reveal that ZAT10 positively regulates the transcriptional activity of some detoxification genes (*NAS1*, *NAS2*, *IRT2*, and *MTP3*) under Cd stress, thereby enhancing Cd tolerance in plants.

### ZAT10 interacts with FER-like iron deficiency-induced transcription and co-regulates the transcriptional expression of *IRT1*

We further performed Y1H assays to test whether these downstream genes are directly regulated by ZAT10. However, it is unfortunate that ZAT10 could not bind to the promoters of these genes in yeast (Supplementary Figure 3), suggesting that ZAT10 may not directly regulate the expression of these genes.

Previous research has shown that FIT is not only a key regulator of iron uptake and homeostasis, but also plays a role in Cd tolerance in plants, as an important factor in the interrelationship between Fe and Cd in plants (Wu et al., 2012). Specifically, FIT interacts with bHLH transcription factors to regulate Fe/Cd uptake genes *IRT1* (Wang et al., 2007; Yuan et al., 2008; Wu et al., 2012), as well as *HMA3*, *MTP3*, *IREG2*, *IRT2*, *NAS1*, and *NAS2*, which are involved in Fe/Cd homeostasis in cells (Wu et al., 2012). Furthermore, a recent study showed that ZAT12, a homolog of ZAT10, negatively regulates *FIT* transcription and directly interacts with FIT proteins to enhance the stability of FIT proteins (Ben Daniel et al., 2016; Le et al., 2016). Therefore, we wondered whether ZAT10 regulates these downstream genes through modulating FIT.

**FIGURE 6:**
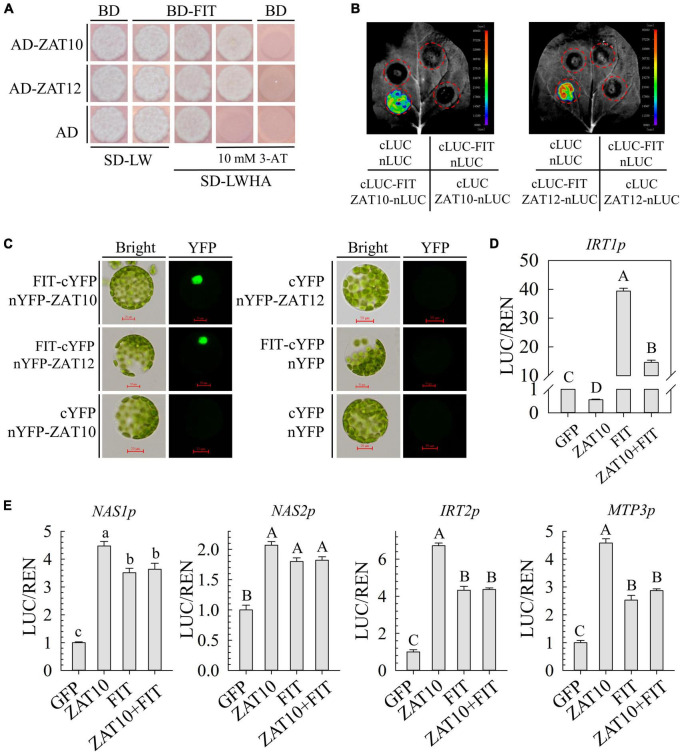
ZAT10 interacts with FER-like iron deficiency-induced transcription and co-regulates the transcriptional expression of *IRT1*. **(A)** ZAT10 interacts with FIT in yeast. 10 mM 3-AT was applied to inhibit the self-activation. ZAT12 was applied as a positive control. **(B)** luciferase complementation imaging assay confirms the interactions between FIT and ZAT10 or ZAT12 in *N. benthamiana* leaves. At least three replicates were observed with similar results. **(C)** BiFC assay confirms the interactions between FIT and ZAT10 or ZAT12 in *Arabidopsis* mesophyll cell protoplasts. At least three replicates were observed with similar results. Bar = 50 μm. **(D)** ZAT10 represses the transcriptional activation of FIT on *IRT1p:LUC* in *Arabidopsis* protoplasts. **(E)** ZAT10 has no effect on the transcription activity of FIT on *NAS1p:LUC*, *NAS2p:LUC*, *IRT2p:LUC*, and *MTP3p:LUC* in *Arabidopsis* protoplasts. Means ± SD, *n* = 3; Significant differences are indicated by letters (lowercase *P* < 0.05 or uppercase *P* < 0.01. Tukey’s multiple comparisons test).

First, we examined the function of ZAT10 in the transcriptional regulation of *FIT* through our transient expression system in *Arabidopsis* protoplasts. The results showed that ZAT10 did not affect the transcriptional activity of *FIT* (Supplementary Figure 4). Next, the yeast two-hybrid assay showed that ZAT10 physically interacted with FIT in yeast cells (Figure 6A). This interaction was further verified by the split luciferase complementation imaging assay (LCI) in *N. benthamiana* leaves and the bimolecular fluorescence complementation assay (BiFC) in *Arabidopsis* protoplasts (Figures 6B,C). ZAT12, which has been previously reported to interact with FIT, was applied as a positive control in these experiments (Figures 6A–C; Ben Daniel et al., 2016; Le et al., 2016). These results suggest that ZAT10 may coordinate with FIT to regulate downstream genes.

Then, transient expression assays in *Arabidopsis* protoplasts were carried out to look into the relationship between ZAT10 and FIT in the transcriptional regulation of downstream genes. The quantification of LUC activity relative to REN showed that the expression of the *IRT1p:LUC* reporter gene was independently suppressed by ZAT10 and induced by FIT (Figure 6D). IRT1p:LUC reporter gene was also induced when ZAT10 and FIT were co-expressed, but to a significantly lower extent than that induced by FIT alone (Figure 6D). Furthermore, the expression of *NAS1p:LUC*, *NAS2p:LUC*, *IRT2p:LUC*, and *MTP3p:LUC* reporter genes was induced by either ZAT10 or FIT (Figure 6E). However, it should be noted that simultaneous expression with ZAT10 and FIT did not further raise the expression levels of these reporter genes; rather, they remained at levels similar to those observed when FIT was expressed alone (Figure 6E).

### Feedback regulation of *ZAT10* expression

**FIGURE 7:**
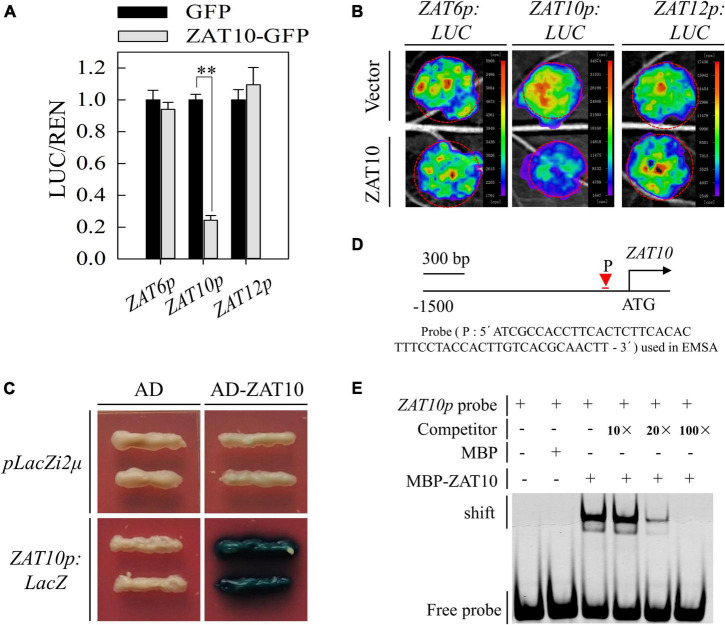
ZAT10 directly binds to its own promoter and represses its expression. **(A)** ZAT10 represses *ZAT10p:LUC* in protoplast transient expression assays. The renilla was used as an internal control. LUC/REN ratio represents the relative activity of the ZAT6, ZAT10, and ZAT12 promoters. Means ± SD, *n* = 3; **P < 0.01, Student’s *t*-test. **(B)** Transient expression assays of luminescence intensity show that ZAT10 represses the transcriptional activity of *ZAT10* promoter in *N. benthamiana* leaves. The *ZAT6p:LUC*, *ZAT10p:LUC*, and *ZAT12p:LUC* were co-infiltrated with empty vector or *ZAT10* overexpression vector in *N. benthamiana* leaves as indicated. Images were taken at 48 h after infiltration. At least three replicates were measured with similar results. **(C)** Yeast one-hybrid assay shows that ZAT10 directly bind to the *ZAT10* promoter. **(D)** Illustration of the probe (P) used in electrophoretic mobility shift assay. **(E)** EMSA analysis shows the direct binding of ZAT10 to the *ZAT10* promoter.

Given that ZAT6, ZAT10, and ZAT12 are conserved in their sequences and their functions may be linked during abiotic stress (Mittler et al., 2006; Shi et al., 2014; Chen et al., 2016), we further tested the effect of ZAT10 on the activity of *ZAT6*, *ZAT10*, and *ZAT12* promoters. Dual-luciferase assay in *Arabidopsis* protoplast showed that ZAT10 acted as a feedback regulator inhibiting the activity of *ZAT10* promoter, while it had no effect on *ZAT6* and *ZAT12* promoter activities (Figure 7A). To verify this result, transient expression assays were carried out. *ZAT6p:LUC*, *ZAT10p:LUC*, or *ZAT12p:LUC* reporter constructs with or without *35S:ZAT10* were infiltrated into *N. benthamiana* leaves. As shown in Figure 7B, only the reporter genes driven by the ZAT10 promoter were significantly repressed by supplementary ZAT10, suggesting that ZAT10 indeed suppresses the expression of *ZAT10*. In addition, the Y1H (Figure 7C) and EMSA (Figure 7D) assays showed that ZAT10 directly bind to the *ZAT10* promoter, further confirming the negative transcriptional regulation of ZAT10 on itself.

## Discussion

Cadmium is a major heavy metal pollutant in soil that is toxic to plants. It severely impairs plant growth and development, and threatens animals and people *via* the food chain. Previous research has shown that there are two different strategies for plants to respond to Cd stress, either by reducing the uptake of Cd by roots, or by chelating and sequestering Cd within cells to reduce the toxicity of Cd. In the present study, we showed that *ZAT10* is up-regulated by Cd exposure (Figure 1), while the *zat10* mutant is more sensitive to Cd stress (Figures 2, 3). Further experiments revealed that ZAT10 represses the transcriptional activation of *IRT1* to reduce Cd uptake in plants (Figure 4), and induces the expression of detoxification genes to reduce Cd toxicity by promoting Cd segregation, leading to enhanced tolerance to Cd (Figure 5). We also demonstrated that ZAT10 interacts with FIT. In addition, we identified a negative feedback regulation of the ZAT10 protein on its own transcriptional activation (Figure 7).

Previous studies have shown that the uptake and accumulation of Cd and a variety of other elements in plants are interrelated (Bao et al., 2010; Zhu et al., 2012; He et al., 2017b; Cheng et al., 2020; Meng et al., 2022). The Fe deficiency condition promotes Cd uptake in different plants (Bao et al., 2010). *IRT1*, regulated by FIT, is a key gene controlling Fe uptake (Yuan et al., 2008; Wang et al., 2013). *IRT1* is induced by Fe deficiency circumstances to improve Fe uptake and also promote the absorption of other metal ions, suggesting that *IRT1* is not only a transporter of Fe but also plays an important role in the uptake of other metals by plants (Vert et al., 2002; He et al., 2017a). Many studies have shown that additional Fe supply under Cd stress significantly inhibits *IRT1* expression, thereby reducing Cd uptake and Cd accumulation and alleviating the suppression of plant root elongation by Cd (Clemens, 2006; Wu et al., 2012; Fan et al., 2014; He et al., 2017a). However, a recent study found that application of Fe increased the expression of *IRT1* under Cd stress and still alleviated the toxicity of Cd to the root system (Meng et al., 2022). Regardless, these results suggest the presence of Fe-Cd uptake competition in plants. Based on these studies, we hypothesized that due to the competition between Cd and Fe in uptake, Cd exposure leads to the activation of Fe deficiency signaling pathways in plants and deficiency-responsive genes, such as *IRT1*, are induced to promote Fe uptake. However, these genes conversely lead to more Cd uptake and exacerbate the toxic effects of Cd on plants. Our findings revealed that ZAT10 down-regulated *IRT1* expression and suppressed the induction level of *IRT1* transcription by FIT (Figures 4, 6D). Thus, we proposed that Cd-induced ZAT10 can reduce Cd uptake by breaking the vicious cycle of “Cd stress-FIT-IRT1 induction-more Cd uptake” to alleviate the Cd hijacking of the Fe transporter. Consistent with this notion, the *zat10* mutant plants displayed a significantly higher Cd accumulation in mutant plants under Cd stress than that in the wild-type plants (Figures 2E,F).

In addition to responding to Cd stress by regulating Cd uptake, plants can also enhance Cd tolerance through detoxification, which involves the translocation and sequestration of Cd in the plant. It has been reported that Cd treatment significantly reduced Fe transport from roots to shoots in plants, suggesting that Cd transport also involves competition with Fe for transport factors (Yao et al., 2018). Previous studies have demonstrated that FIT coordinates with bHLHs (bHLH38, bHLH39, bHLH100, and bHLH101) to activate the transcription of *NAS1* and *NAS2*, which regulate the synthesis of important metal chelator nicotiananamine (NA). On the other hand, FIT promotes the sequestration of heavy metals in vacuoles and vesicles in *Arabidopsis* by regulating *HMA3*, *MTP3*, *IREG2*, and *IRT2* (Yuan et al., 2008; Wu et al., 2012; Wang et al., 2013). In this study, we also found that ZAT10 activated the transcription of *NAS1*, *NAS2*, *MTP3*, and *IRT2* under Cd stress (Figure 5), indicating ZAT10 could enhance the Cd chelation and sequestration. Taken together, ZAT10 can simultaneously suppress the expression of Cd uptake related genes and enhance the expression of Cd segregation related genes, indicating that its regulatory mechanisms may differ among genes with different functions.

ZAT10 does not bind to the promoter of these downstream genes in yeast (Supplementary Figure 3), suggesting that ZAT10 may indirect regulate downstream genes through other proteins. Our investigation revealed that *ZAT10* interacts with FIT (Figures 6A–C), and all of the ZAT10-regulated downstream genes identified in this study are also regulated by FIT (Figures 6D,E). However, the regulatory relationship between them remains unclear. For the Cd uptake related gene *IRT1*, additional ZAT10 could suppress the expression induced by FIT (Figure 6D). In contrast, for Cd segregation-related genes, the simultaneous action of ZAT10 and FIT still showed similar levels of activation as FIT alone did, suggesting that although ZAT10 interacts with FIT, additional ZAT10 does not affect the regulation of these genes by FIT (Figure 6E). One possible explanation is that the interaction between FIT and ZAT10 affects the regulation of downstream genes by FIT. However, based on our current data, we cannot exclude the possibility that *ZAT10* regulates downstream genes through other pathways independent of FIT or that a combination of these two mechanisms exists for different target genes.

Moreover, we identified two negative feedback regulatory pathways for *ZAT10*. First, we found that Cd stress induces H_2_O_2_ accumulation in plants, and the accumulated H_2_O_2_ negatively regulates *ZAT10* expression (Figure 3). Similarly, previous studies reported that H_2_O_2_ decreases the Fe-deficiency-induced *FIT* expression and suppresses the induction of *FIT* in *zat12* mutant plants, but induces more FIT and ZAT12 protein accumulation (Le et al., 2016). These results indicate that H_2_O_2_ is an important signal factor in the regulatory pathway of metal ion uptake and transport in plants. Secondly, the expression of *ZAT10* was induced by Cd stress, and ZAT10 protein could directly bind to the promoter of *ZAT10* to suppress its transcription as the feedback regulation loop (Figure 7). These two negative regulatory pathways protect plants from the over-response to Cd stress and may explain why the *ZAT10* expression decreased after reaching a peak at 6 h of Cd treatment (Figure 1D). Furthermore, homology analysis and sequence alignment showed that ZAT10 in different crops, such as soybean, rice, maize, and pepper, are highly homologous to At ZAT10 (Supplementary Figure 1), suggesting that their functions may be highly conserved. Therefore, *ZAT10* may have potential applications in the improvement of Cd tolerance in crops.

**FIGURE 8:**
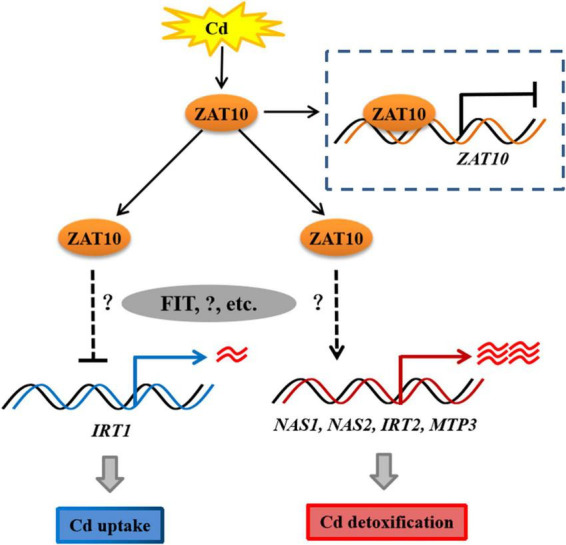
A schematic model depicting the role of ZAT10 in regulating cadmium tolerance in *Arabidopsis*. *ZAT10* is induced by Cd stress, which in turn suppresses its own expression. ZAT10 negatively regulate the expression of the Cd uptake gene *IRT1*, leading to a reduction in Cd uptake in plants. ZAT10 also promotes the expression of the Cd detoxification genes *NAS1*, *NAS2*, *IRT2*, and *MTP3*, to enhance the detoxification of Cd in plants. Dashed lines with a question mark denote unclear regulatory mechanisms.

In summary, in this study, we demonstrated that *ZAT10* has dual functions in plants in response to Cd stress. ZAT10 is involved in the regulation of both key genes for Cd uptake and those for Cd chelation and sequestration, thus enhancing the resistance of plants to Cd stress. The data presented here show that ZAT10 interacts with FIT, but the regulatory relationship between them on downstream genes remains unclear, hence further investigation is merited. In addition, we reported a negative feedback regulation loop of *ZAT10*. Based on our results, we proposed a brief model of the regulatory mechanism by which *ZAT10* regulates plant responses to Cd stress (Figure 8). Taken together, our results provide new perspectives into the molecular function of *ZAT10* in plants under Cd stress and *ZAT10* is a potential candidate gene for improving cadmium tolerance in plants.

## Data availability statement

The original contributions presented in this study are included in the article/Supplementary material, further inquiries can be directed to the corresponding author.

## Author contributions

FD and XL designed the research. FD, YL, JL, and XL performed the experiments. FD, YW, SD, and XL wrote the manuscript. All authors analyzed the data, read and approved the manuscript.

## Footnotes

1
https://abrc.osu.edu/

2
https://www.arabidopsis.org/

## Supplementary material

Supplementary Figure 1 Phylogenetic analysis and sequence alignment of ZAT10 in different plant species.

Supplementary Figure 2 Expression of *ZIP3*, *ZIP4*, *ZIP5*, *ZIP9*, *IREG1*, *IREG2*, and *HMA3* in the wild-type and *zat10* mutant plants under cadmium stress.

Supplementary Figure 3 Yeast one-hybrid assay showing that ZAT10 does not bind to the promoters of *IRT1*, *NAS1*, *NAS2*, *IRT2*, and *MTP3*.

Supplementary Figure 4 ZAT10 has no effect on the activity of *FITp:LUC* in protoplast transient expression assays.

Supplementary Table 1 List of the primers used in this study.
