# Supplementary material for: ZAT10 plays dual roles in cadmium uptake and detoxification in Arabidopsis
Source: Front Plant Sci. 2022 Aug 30;13:994100. doi: 10.3389/fpls.2022.994100 (PMC9468636; doi:10.3389/fpls.2022.994100)
Supplement: Supplementary Figure 1 — Phylogenetic analysis and sequence alignment of ZAT10 in different plant species. [file Data_Sheet_1.pdf]

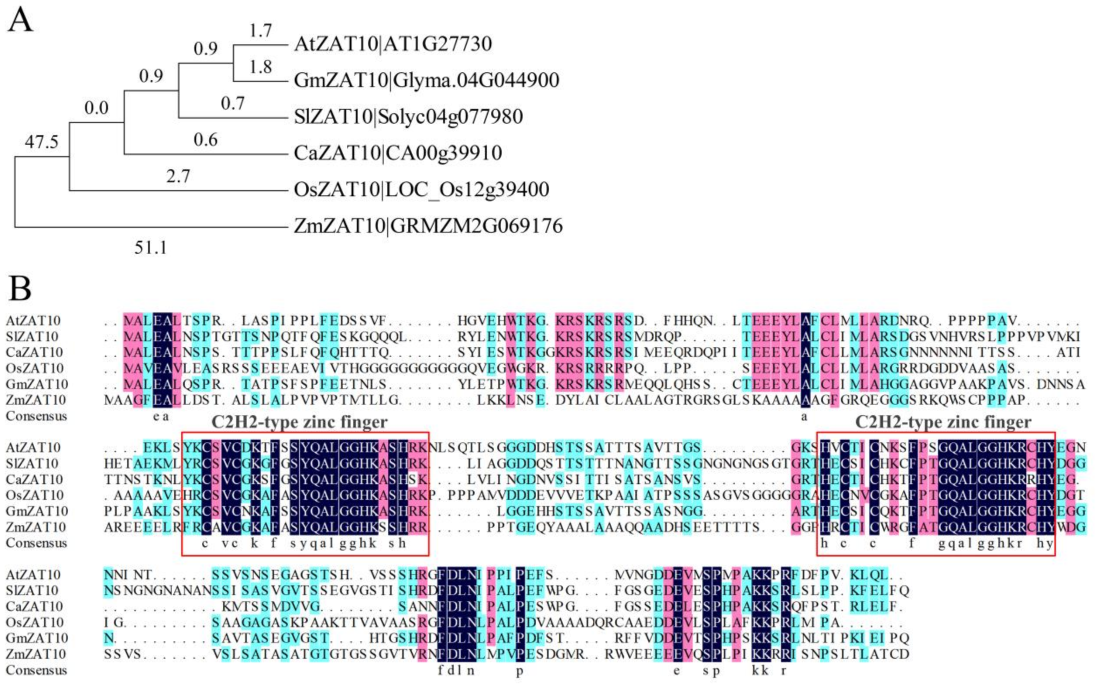

Supplementary Figure 1. Phylogenetic analysis and sequence alignment of ZAT10 in different plant species.

(A) Phylogenetic tree of ZAT10 in different plant species.

(B) Amino acid sequence alignment of ZAT10 in different plant species. The two conserved C2H2-type zinc finger domains are indicated.

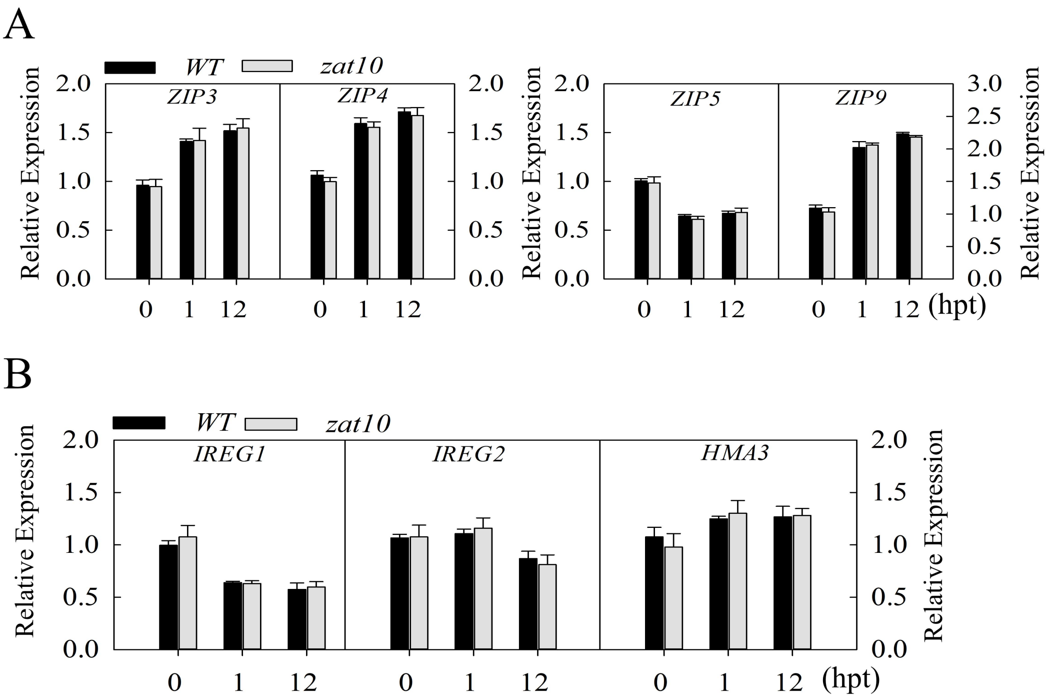

Supplementary Figure 2. Expression of *ZIP3*, *ZIP4*, *ZIP5*, *ZIP9*, *IREG1*, *IREG2*, and *HMA3* in the wild-type and *zat10* mutant plants under Cd stress.

Relative expression levels of these genes were analyzed by qRT-PCR in the wild-type and *zat10* mutant plants at 0, 1 and 12 h post treatment (hpt) with 30 μM $CdSO_4$. Means ± SD, n = 3.

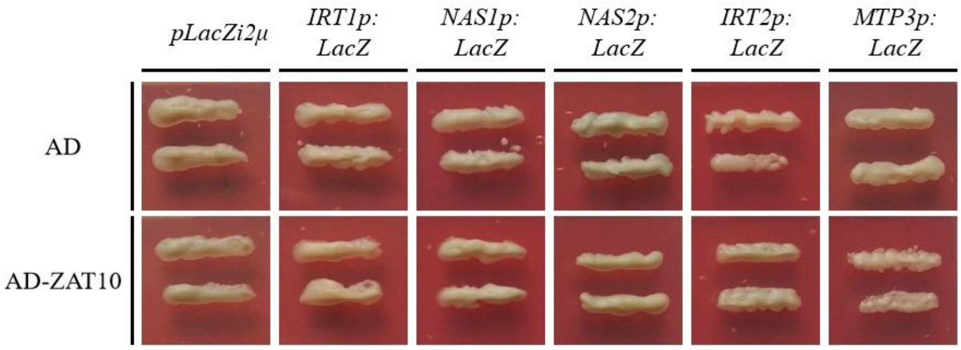

Supplementary Figure 3. Yeast one-hybrid assay showing that ZAT10 does not bind to the promoters of *IRT1*, *NAS1*, *NAS2*, *IRT2* and *MTP3*.

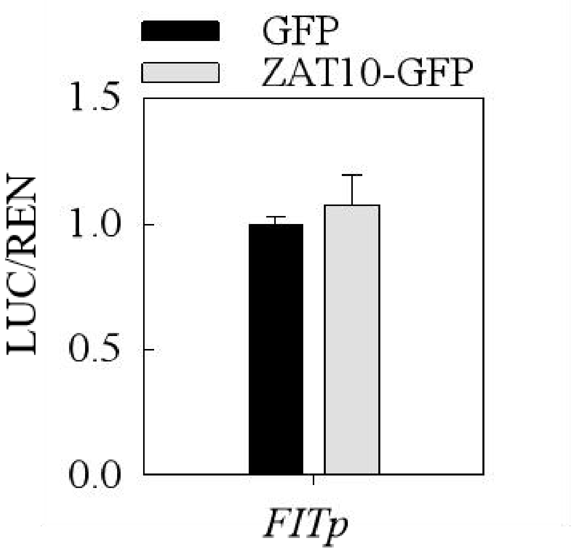

Supplementary Figure 4. ZAT10 has no effect on the activity of *FITp:LUC* in protoplast transient expression assays.

The protoplasts were transfected with 20 µg *FITp:LUC* plasmid and 20 µg *p19-GFP* or 20 µg *p19-ZAT10-GFP* plasmids as indicated. The transfected protoplasts were harvested for LUC and REN measurements after 10 h incubation in WI buffer. The REN is used as an internal control. LUC/REN ratio represents the relative activity of the *FIT* promoter. Means ± SD, n = 3.
