# Supplementary material for: ZAT10 plays dual roles in cadmium uptake and detoxification in Arabidopsis
Source: Front Plant Sci. 2022 Aug 30;13:994100. doi: 10.3389/fpls.2022.994100 (PMC9468636; doi:10.3389/fpls.2022.994100)
Supplement: Supplementary Table 1 — List of the primers used in this study. [file Table_1.PDF]

Supplementary Table 1. List of the primers used in this study

Primer Name	Forward (5′-3′)	Reverse(5′-3′)
**Primers used for DNA constructs**		
ZAT6 (CDS)-Zero	ggatcttccagagatATGGCACTTGAAACTCTTAC	ctgccgttcgacgatTTAGGGTTTCTCCGGGAAGTCA
ZAT10 (CDS)-Zero	ggatcttccagagatATGGCGCTCGAGGCTCTT	ctgccgttcgacgatTTAAAGTTGAAGTTTGACC
ZAT12 (CDS)-Zero	ggatcttccagagatATGGTTGCGATATCGGAGAT	ctgccgttcgacgatTCAATAAACTGTTCTTCCAAG
ZAT10 (genome)-Zero	ggatcttccagagatTTTATCATGTATTACAAGTT	ctgccgttcgacgatACTAGAACGTTCTTGATTAT
p1300-ZAT10	catgattacgaattcgagctcTTTATCATGTATTACAAGTT	cttgcatgcctgcaggtcgacACTAGAACGTTCTTGATTATGC
**Primers used for LUC/REN , Y1H, and EMSA**		
ZAT10-GFP	gacaagcttggtacctctagaATGGCGCTCGAGGCTCTTAC	gcccttgctcaccatactagtAAGTTGAAGTTTGACCGGAAA
FIT-GFP	gacaagcttggtacctctagaATGGAAGGAAGAGTCAACGC	gcccttgctcaccatactagtAGTAAATGACTTGATGAATTCAAA
ZAT10-62SK	cgctctagaactagtggatccATGGCGCTCGAGGCTCTT	gtcgacggtatcgataagcttTTAAAGTTGAAGTTTGACC
ZAT6-0800	gtcgacggtatcgataagcttTAATCCATGATTTAGAACA	cgctctagaactagtggatccTATCTTGAAGACTAGCTAC
ZAT10-0800	gtcgacggtatcgataagcttTTTATCATGTATTACAAGTT	cgctctagaactagtggatccTAAGTTAAAGATTCTGAGG
ZAT12-0800	gtcgacggtatcgataagcttGGAAGTGTGTAAGTATAATA	cgctctagaactagtggatccTTTTCTTCTGATGATGATGA
IRT1-0800	gtcgacggtatcgataagcttAATTTGTGTCAATATCTGATTTGGT	cgctctagaactagtggatccTTTTTTTTTTTTCTTTTTTCTTTTG
ZIP3-0800	gtcgacggtatcgataagcttTTCATCTTTTCGATTTCTTAA	cgctctagaactagtggatccAATCTCTATCTTATTTTAAAA
ZIP4-0800	gtcgacggtatcgataagcttAAATTGTTTTCTGAATCAGG	cgctctagaactagtggatccTGTCTCGGGAATGATCTGCA
ZIP5-0800	gtcgacggtatcgataagcttACTCGTGATTTTTAAAGCTC	cgctctagaactagtggatccCTTATCGATTAGGGTTTGAA
ZIP9-0800	gtcgacggtatcgataagcttCTTTTATCGAATCTGAAAAT	cgctctagaactagtggatccAGCTGCGAACTTGAGGGTAA
IRT2-0800	gtcgacggtatcgataagcttTTCTTAACTTAGCAACACAC	cgctctagaactagtggatccTAGTATTGAGATTGTTTTAT
IREG1-0800	gtcgacggtatcgataagcttCCCATTTCCTCCATGGACAG	cgctctagaactagtggatccTTTCTGCTGGAAAGTCTCG
IREG2-0800	gtcgacggtatcgataagcttCAAAATCACATCCCTAAACG	cgctctagaactagtggatccTTCTTCTGACTACTTTGATT
NAS1-0800	gtcgacggtatcgataagcttAAGCAAACGAAACAATCAGGA	cgctctagaactagtggatccATCGACACTATGAGGTTATTTC

NAS2-0800	gtcgacggtatcgataagcttGATTCGGATATTATAGCGAGTTC	cgctctagaactagtggatccGTCGACACTATGAAGAGAGAA
HMA3-0800	gtcgacggtatcgataagcttGATGAGTGAAGAAGGTGAA	cgctctagaactagtggatccCGTTGAGCTTGAGATCTCGG
MTP3-0800	gtcgacggtatcgataagcttTTAGGGGCTTCATCTGGTGG	cgctctagaactagtggatccACCATTCTGGCAAACTCTTTAT
ZAT10-JG45	tgcctctcccgaattcATGGCGCTCGAGGCTCTT	tccaaagcttctcgagTTAAAGTTGAAGTTTGACC
ZAT10P-pLaczi	tattggatcggaattcCATACGAGTACCATCCATGAC	gagcacatgcctcgagGATTCTGAGGATTTCTTGTCTC
ZAT10-*Cy5*-EMSA	ATCGCCACCTTCACTCTTCACACTTTCCTACCACTTGTCACGCAACTT	AAGTTGCGTGACAAGTGGTAGGAAAGTGTGAAGAGTGAAGGTGGCGAT
**Primers used for Y2H, LCI, and BiFC**		
BD-FIT	catggaggccgaattcATGGAAGGAAGAGTCAACGC	gcaggtcgacggatccAGTAAATGACTTGATGAATTCAAA
AD-ZAT10	gccatggaggccagtgaattcATGGCGCTCGAGGCTCTTAC	cagctcgagctcgatggatccAAGTTGAAGTTTGACCGGAAA
AD-ZAT12	gccatggaggccagtgaattcATGGTTGCGATATCGGAGAT	cagctcgagctcgatggatccATAAACTGTTCTTCCAAGC
cLUC-FIT	gcgtcccggggcggtaccATGGAAGGAAGAGTCAACGC	aaagctctgcaggtcgacTCAAGTAAATGACTTGATGAATTC
ZAT6-nLUC	ggacgagctcggtaccATGGCACTTGAAACTCTTAC	gtacgagatctggtcgacGGGTTTCTCCGGGAAGTCA
ZAT10-nLUC	ggacgagctcggtaccATGGCGCTCGAGGCTCTTAC	gtacgagatctggtcgacAAGTTGAAGTTTGACCGGAAA
ZAT12-nLUC	ggacgagctcggtaccATGGTTGCGATATCGGAGAT	gtacgagatctggtcgacATAAACTGTTCTTCCAAGC
cYFP-FIT	tcagcagtcgaagagcATGGAAGGAAGAGTCAACGC	ttagcgtgtgaagagcTCAAGTAAATGACTTGATG
ZAT10-nYFP	tcagcagtcgaagagcATGGCGCTCGAGGCTCTTAC	ttagcgtgtgaagagcAAGTTGAAGTTTGACCGGA
ZAT12-nYFP	tcagcagtcgaagagcATGGTTGCGATATCGGAGAT	ttagcgtgtgaagagcATAAACTGTTCTTCCAAGC
**Primers used for *zat10* mutant identification**		
LB1.3	ATTTTGCCGATTTCGGAAC	
ZAT10-LP	TATTTTGTAAGGCGGCATCAG	
ZAT10-RP	AAGTCAAACCGAGGCTTCTTC	
**Primers used for qRT-PCR**		
AtZAT10 (AT1G27730)	GTCAACGGAGACGACGAAGTCA	AGTTGTAGTGGTGTCACTTTATGCT
AtIRT1 (AT4G19690)	GCCCCGCAAATGATGTTACC	TCCAATGACCACCGAGTGAA
AtZIP3 (AT2G17790)	CTTGGTGCTGGCCTCATGTCTC	TCACAAATCACGCAAGCAAGCA
AtZIP4 (AT1G10970)	GATCTTCGTCGATGTTCTTTGG	TGAGAGGTATGGCTACACCAGCAG

AtZIP5 (AT1G05300)	AGTGGACTTTCTCGCAGCAGAT	TGGTAGGCCCATTTAAGCCCAT
AtZIP9 (AT4G33020)	TAGGCTTGGACAGTGGTGTGAGG	GCGAGAAGGAGAGGACGTATGGT
AtIRT2 (AT4G19680)	TGCATCCTCCAGGCAGATTT	GACCCCATGAACTCGGTAGC
AtIREG1 (AT2G38460)	ATGAATCACAGCCACAAAACC	CCCTCGACCCATTGCCCCAC
AtIREG2 (AT5G03570)	TCACCTCTCAGAACCGGAGT	TAGAAGCAGCAACTCCAGCC
AtNAS1 (AT5G04950)	TGCTTACCCACGGATACAAA	ATCGGACCCGAACCCACGAA
AtNAS2 (AT5G56080)	ACACTTTTCAGCAATCTTAGGC	CTTTGGTCGGGACATGGGTT
AtHMA3 (AT4G30120)	CCAGCTGTTGTTGTGTCAGC	GACCACAGGGACAACCACTT
AtMTP3 (AT3G58810)	CCACGAGAGATTGACCCGAC	TATCTGCCTCTGCCTCAGGT
AtUBQ10 (AT4G05320)	TCCTCAGGCTCCGTGGTGGT	AGCCTCTGCTGGTCCGGAGG
